# Gunshot Wound Tracheal Injury and Endotracheal Tube Perforation Into Soft Tissue Pocket With Ventilation Solely Through Murphy’s Eye: A Case Report

**DOI:** 10.7759/cureus.97963

**Published:** 2025-11-27

**Authors:** Michael Selby, Kolos K Nagy, Daniel Brodie, Kelan Nesbitt

**Affiliations:** 1 School of Medicine, Texas Tech University Health Sciences Center, Lubbock, USA; 2 Anesthesiology, Texas Tech University Health Sciences Center, Lubbock, USA

**Keywords:** airway mangement, difficult ventilation, gun shot wound, murphy's eye, placement of endotracheal tube (ett)

## Abstract

A patient arrived at the emergency department by ambulance with a gunshot wound to the chest. Concern for tracheal injury was confirmed by imaging, also revealing perforation of the endotracheal tube through the tracheal defect. In the operating room (OR), a fiberoptic bronchoscope was used to visualize the airway, revealing that ventilation was solely occurring through Murphy’s eye, which was incidentally lined up with the tracheal defect. A fiberoptic scope was used to reposition the endotracheal tube distal to the injury, securing the airway. This case demonstrates the utility of Murphy’s eye and the indispensable role of fiberoptic bronchoscopy in airway navigation.

## Introduction

An endotracheal tube (ETT) is a tube commonly made of polyvinyl chloride that is used to ventilate the lungs with oxygen and other gases, facilitating gas exchange. It has five components: the tube, cuff, bevel, connector, and Murphy’s eye. The cuff is an inflatable portion of the ETT located proximal to its distal end. When inflated, the cuff produces a seal against the tracheal wall, ensuring secure placement and protection from aspiration. The bevel is the angled portion at the distal end of the ETT, used to facilitate its placement. The connector is its most proximal component and is attached to a circuit, allowing mechanical ventilation with the anesthesia machine [1]. Murphy’s eye is an opening on the lateral side of the tube just proximal to the bevel and serves as a safety mechanism in case the distal tip of the ETT is obstructed [2].

The utility of Murphy’s eye has been debated due to several potential drawbacks, including its small size, allowing only limited gas flow; its unilateral placement, making the flow distribution sensitive to its position; and its potential impact on the reliability of auscultation for detecting endobronchial intubation [2,3]. Murphy’s eye serves as a safety mechanism if the distal end of the endotracheal tube (ETT) is obstructed by mucus, the tracheal wall, or hematoma. To the best of our knowledge, there is no existing literature on temporarily adequate ventilation through Murphy's eye in an ETT slid outside of the trachea through a defect from a gunshot wound (GSW) [1,4]. Though Murphy’s eye allows minimal ventilation when compared to the distal opening of an ETT, the positioning of Murphy's eye directly over the tracheal injury in the patient presented here allowed adequate ventilation until arrival at the operating room (OR). Had Murphy's eye not aligned with the tracheal defect, ventilation would have been impossible during the transport and evaluation of this patient. Thus, the patient would have been at high risk of demise. In the OR, the anesthesiology team used a fiberoptic bronchoscope to successfully reposition the ETT distal to the injury, securing the airway and allowing surgical repair to proceed.

While Murphy’s eye and the fiberoptic bronchoscope may not be necessary in many routine anesthesia cases, their serendipitous interplay in this case makes a compelling argument for the utility of Murphy’s eye and the indispensable role of fiberoptic intubation in precarious airway management [3]. In this case report, we highlight the utility of the Murphy’s eye in an acute traumatic airway and demonstrate how a fiberoptic bronchoscope was used to successfully reposition an ETT when direct visualization was limited. Herein, we aim to emphasize the necessity of both the Murphy’s eye and fiberoptic bronchoscopy in managing challenging airways, supporting their continued, indispensable role in modern anesthesia practice.

## Case presentation

**Figure 1:**
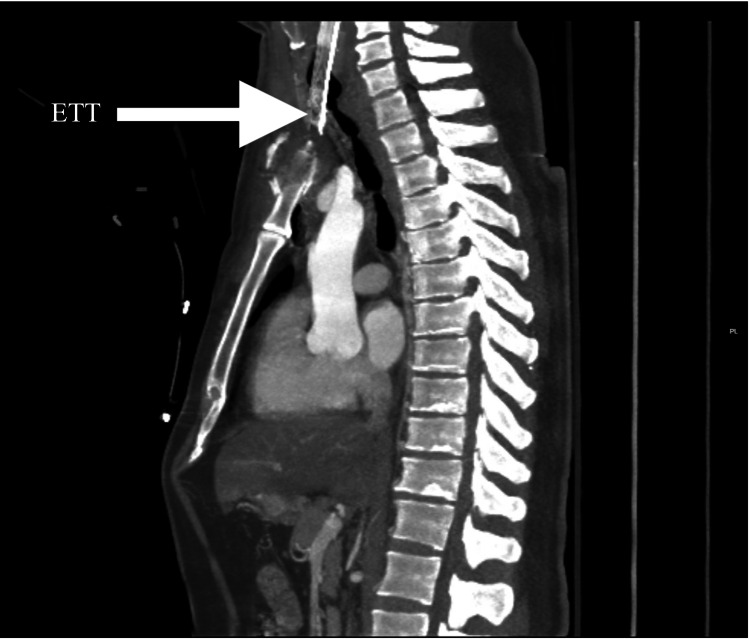
Sagittal CT chest showing anterior perforation of endotracheal tube (ETT) through the tracheal injury

**Figure 2:**
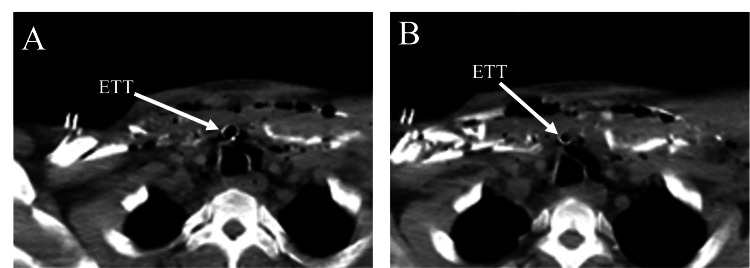
Axial CT chest showing perforation of endotracheal tube (ETT) through the tracheal injury

A 35-year-old male presented to the emergency department (ED) with a GSW to the upper mid-thorax. Past medical history and social history were unknown. The patient was intubated in the field by emergency medical services (EMS), and upon arrival, he was saturating in the mid-80s on pulse oximetry. The patient was tachycardic, but blood pressure was within normal limits. The Focused Assessment with Sonography in Trauma (FAST) exam was negative, and he was subsequently sent for computed tomography (CT) imaging. CT demonstrated a tracheal injury with perforation of the ETT anteriorly from the trachea through a defect, as well as bilateral clavicular fractures, sternal fracture, soft tissue swelling, and subcutaneous emphysema (Figures 1, 2). The patient was immediately transferred to the OR for an airway exam and neck exploration.

Operating room course

In the OR, the patient was placed in the supine position and connected to the anesthesia machine circuit. He arrived sedated with a fentanyl infusion and was paralyzed with 100 mg rocuronium once positioned and connected to the circuit. The patient was placed on pressure control-volume guaranteed (PCV-VG) ventilation, and limited ventilation became evident. Peak pressures were maxed at the safety limit of 35 cm H2O with tidal volumes set at 200 mL. End-tidal pCO2 remained in the 60-70 mmHg range, indicating significant airway resistance and inadequate minute ventilation, despite favorable pO2 saturation >90% on 100% oxygen. Initial arterial blood gas (ABG) confirmed elevated pCO2 and mixed respiratory and lactic acidosis with a pH of 7.19.

**Figure 3:**
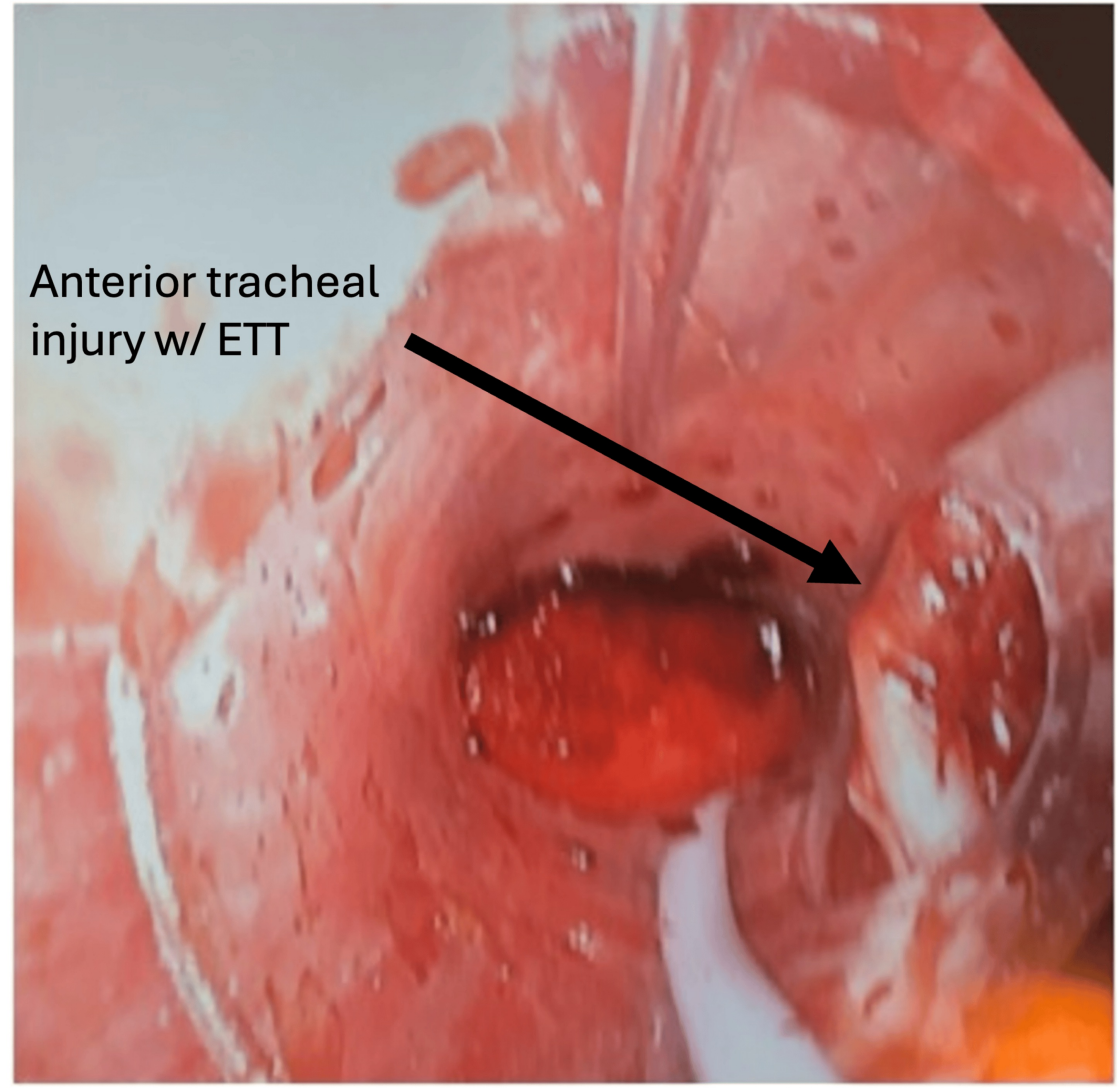
Fiberoptic bronchoscope view of Murphy’s eye lined up with the injury site allowing for ventilation.

After confirming mechanical connections and the absence of kinks in the ETT, bronchoscopy was performed with a fiberoptic scope via the ETT. The proximal trachea was seen on initial entry, but further advancement of the scope revealed the ETT exiting the trachea anteriorly through the tracheal injury. The distal end of the tube was positioned in a tract of tissue anterior to the trachea, likely created by the blast injury. Tracheal rings proximal and distal to the transection were visible posterior to the ETT, revealing that Murphy’s eye was positioned perfectly in line with the defect, and that ventilation was likely occurring through this path (Figure 3). The distal end of the ETT was sealed in the tissue tract, further suggesting that all ventilation was occurring through Murphy’s eye.

After discussion with the surgical team, a surgical airway was prepared before attempting to reposition the ETT in case the airway was lost and emergent tracheostomy was necessary. A soft tissue layer over the trachea was noted to be intact, but the ETT was palpable underneath the fourth tracheal ring, indicating anterior tracheal injury and ETT perforation. This layer was left intact to maintain an airtight seal while repositioning was attempted. After satisfactory access to the trachea was achieved, the ETT was retracted under visualization with the fiberoptic scope. As Murphy’s eye moved proximal to the injury, ventilation ceased, further reaffirming that ventilation was solely occurring through Murphy’s eye. Once the ETT was retracted proximal to the injury, the scope was advanced past the injury into the distal trachea. The ETT was then advanced over the scope. The scope was retracted to visualize the cuff, and after confirmation of the cuff’s position distal to the injury, it was inflated. Ventilation parameters immediately normalized with peak pressures <20 and tidal volumes of 500 mL, accurately reflecting the ventilator settings. End-tidal CO2 immediately began to normalize with adequate ventilation. The new ETT position was confirmed intraoperatively under direct visualization after incising the soft tissue layer previously left intact.

An esophagogastroduodenoscopy was performed to rule out esophageal injury. There was mucosal irritation 23-19 cm distal to the second molar tooth, indicating a substantial blast effect on the esophagus. No other serious injuries were seen. The patient was transported to the surgical intensive care unit, where he was intubated, sedated, and remained stable without vasopressors. ABG indicated significant improvement in acidosis with normalized pCO2 and pH, down-trending lactate, and hemoglobin trending near 11 g/dL.

## Discussion

The ETT is a tube commonly made of polyvinyl chloride that is used to ventilate the lungs with oxygen and other gases, facilitating gas exchange. It has five components: the tube, cuff, bevel, connector, and Murphy’s eye. The cuff is an inflatable portion of the ETT located proximal to its distal end. When inflated, the cuff produces a seal against the tracheal wall, ensuring secure placement and protection from aspiration. The bevel is the angled portion at the distal end of the ETT, used to facilitate its placement. The connector is its most proximal component and is attached to a circuit, allowing mechanical ventilation with the anesthesia machine [3]. Murphy’s eye is an opening on the lateral side of the tube just proximal to the bevel and serves as a safety mechanism in case the distal tip of the ETT is obstructed [1]. This case is among the very few to describe adequate ventilation solely occurring through Murphy’s eye, presumably for a significant amount of time from the field until the patient arrived at the OR. Murphy’s eye, positioned perfectly at the level of the tracheal defect, is an unparalleled demonstration of its purpose. 

Fiberoptic bronchoscopy is considered the gold standard of navigation in difficult airways [5]. Frank J. Murphy, one of the first physicians to popularize fiberoptic intubation, described the indications for the use of a flexible fiberoptic bronchoscope, including special patient groups, confirmation of the position of airway devices, and training [6]. A fiberoptic scope consists of a camera, light source, and remote control that feeds an image to a screen. The remote control is equipped with a thumb-controlled lever that allows manipulation of the direction of the scope by flexion and extension near the camera on the distal tip, and rotation is performed manually. In anesthesiology, a fiberoptic scope can be used to aid in difficult intubation, examination of abnormal laryngeal and tracheal anatomy, double-lumen tube or bronchial blocker placement for single lung ventilation, or diagnostic and therapeutic bronchoscopy [7]. Frequently encountered difficult airways that indicate the use of a fiberoptic scope include previous difficult intubation, hoarse voice, hematoma of vocal cords, airway masses, poor neck mobility, and infection [5]. 

In the setting of a difficult airway, an ETT can be pre-loaded onto a fiberoptic scope and placed by the Seldinger technique after advancing the scope into the desired position in the trachea or bronchus. The fiberoptic scope can then be used to confirm the desired tube position [7,8]. Fiberoptic bronchoscopy allows for the navigation of peculiar airways where the margin of error is slim to none, as in this case. Had it not been available, the necessity of surgical airway creation would have substantially increased.

While fiberoptic bronchoscope navigation has been described for tracheal perforations and gunshot wounds, they are uncommon, and little to no literature exists similar to the presentation of this patient. Fiberoptic scopes provide physicians with a potent tool for managing difficult airways, but proper training and technique are critical [5]. In this case, the ETT perforation was promptly recognized, and the anesthesia and surgical teams were well prepared to deal with the situation. Without proper preparation, communication, and technique, a surgical airway may have been necessary, increasing the risk of complications [9].

## Conclusions

This case highlights the benefits of Murphy’s eye in a challenging circumstance where the distal tip of the ETT was occluded, emphasizing its role as a backup ventilation route. The skillful use of the fiberoptic scope to reposition the ETT demonstrates its effectiveness as an indispensable tool for airway management. To the best of our knowledge, this case is the first to describe a patient with a transtracheal GSW with a perforated ETT where ventilation was only possible because Murphy’s eye incidentally lined up with the tracheal defect. Future reports should further characterize similar airway injuries to clarify the conditions under which Murphy’s eye ventilation is feasible and to guide standardized approaches for fiberoptic-assisted tube repositioning.
